# The effect of fear of falling on vestibular feedback control of balance

**DOI:** 10.14814/phy2.13391

**Published:** 2017-09-28

**Authors:** Jonathan L. A. de Melker Worms, John F. Stins, Peter J. Beek, Ian D. Loram

**Affiliations:** ^1^ Cognitive Motor Function research group School of Healthcare Science Manchester Metropolitan University Manchester United Kingdom; ^2^ Department of Human Movement Sciences Faculty of Behavioural and Movement Sciences Vrije Universiteit Amsterdam Amsterdam Movement Sciences Amsterdam The Netherlands

**Keywords:** fear of falling, full‐body kinematics, galvanic vestibular stimulation, sensorimotor control, vestibular system

## Abstract

Vestibular sensation contributes to cervical‐head stabilization and fall prevention. To what extent fear of falling influences the associated vestibular feedback processes is currently undetermined. We used galanic vestibular stimulation (GVS) to induce vestibular reflexes while participants stood at ground level and on a narrow walkway at 3.85 m height to induce fear of falling. Fear was confirmed by questionnaires and elevated skin conductance. Full‐body kinematics was measured to differentiate the whole‐body centre of mass response (CoM) into component parts (cervical, axial trunk, appendicular short latency, and medium latency). We studied the effect of fear of falling on each component to discern their underlying mechanisms. Statistical parametric mapping analysis provided sensitive discrimination of early GVS and height effects. Kinematic analysis revealed responses at 1 mA stimulation previously believed marginal through EMG and force plate analysis. The GVS response comprised a rapid, anode‐directed cervical‐head acceleration, a short‐latency cathode‐directed acceleration (cathodal buckling) of lower extremities and pelvis, an anode‐directed upper thorax acceleration, and subsequently a medium‐latency anode‐directed acceleration of all body parts. At height, head and upper thorax early acceleration were unaltered, however, short‐latency lower extremity acceleration was increased. The effect of height on balance was a decreased duration and increased rate of change in the CoM acceleration pattern. These results demonstrate that fear modifies vestibular control of balance, whereas cervical‐head stabilization is governed by different mechanisms unaffected by fear of falling. The mechanical pattern of cathodal buckling and its modulation by fear of falling both support the hypothesis that short‐latency responses contribute to regulate balance.

## Introduction

Fear of falling is known to influence human balance (Stins et al. 2011; Tersteeg et al. 2012; Osler et al. 2013). When fearful, movements become more cautious and joint stiffness tends to increase (Adkin et al. 2002; Tersteeg et al. 2012; Osler et al. 2013; Young and Mark 2015). Studies of fall risk in the elderly have shown associations between cognitive motor measures (e.g., concern about falling and poor executive function) and physiological measures of impaired balance (Delbaere et al. 2010; Hadjistavropoulos et al. 2011). From a healthy aging perspective there is a need to understand the mechanisms relating fear of falling to balance and mobility in the elderly. In addition, it has been proposed that anxiety increases sensitivity to self‐motion through noradrenergic and serotonergic input to the vestibular nuclei (Balaban 2002). Therefore, we focus in this study on the vestibular contributions to human balance and the potential interplay with fear of falling.

As evidenced by a recent crosstalk debate (van Dieen et al. 2015; Horslen et al. 2015a,b; Reynolds et al. 2015a,b), it is currently controversial whether fear of falling influences the vestibular control of balance. Bipolar binaural Galvanic Vestibular Stimulation (GVS) is a frequently employed method to study vestibular balance reflexes (Fitzpatrick and Day 2004). Cutaneous electrical stimulation at the mastoid processes stimulates the vestibular nerves and creates erroneous feedback of roll rotation. This elicits a lateral body sway response toward the anode electrode. A paradigm of standing at height on a 22‐cm narrow walkway to evoke fear of falling, combined with GVS, has shown that fear of falling might differentially affect the feedforward and feedback components of the vestibular‐evoked balance response (Osler et al. 2013). Given sufficient time to integrate proprioception of movement with vestibular sensation, vestibular‐evoked sway is strongly arrested at height compared to ground. However, kinematic data of head and torso showed that fear had no measureable effect on the initial (0–800 msec) vestibular‐evoked balance response. In contrast, using a similar height paradigm, Horslen et al. (2014) have shown increased gain in the initial vestibular reflex response. However, in their study ground reaction force (GRF) data were used to assess balance responses and a different stimulation paradigm was employed (SVS, stochastic vestibular stimulation) to elicit vestibular balancing reflexes.

Vestibular information is used within a variety of mechanisms related to balance. Pertinent to this study, vestibular sensory feedback is used to regulate head orientation through the vestibulocolic reflex (VCR) and to regulate balance through responses that control movement of the whole‐body CoM. It is possible that fear of falling has differential effects on these vestibular responses, which have different onset latencies to GVS implying distinct neural pathways. Indeed, researchers examining fear of falling effects in neck, axial, and upper limb muscle groups have found differences from leg responses (Naranjo et al. 2015, 2016). Furthermore, extant literature indicates that appendicular reflexes (upper and lower extremities) are governed by different mechanisms than axial (e.g., cervical) reflexes. For example, appendicular vestibular reflexes are task and posture dependent, whereas axial reflexes are less dependent on task and posture (Forbes et al. 2014, 2015, 2016).

EMG data have been used to reveal the latency of vestibular responses and thereby establish the neural pathways that could be involved. For example, the VCR has a latency of approximately 8–10 msec (Watson and Colebatch 1998; Forbes et al. 2014). When recording lower limb muscles during upright standing, short‐ and medium‐latency vestibular balancing responses were found. The onset of these short‐latency responses ranged from 42 to 65 msec, and for medium‐latency from 98 to 120 msec post‐GVS onset (Britton et al. 1993; Fitzpatrick et al. 1994; Ali et al. 2003; Fitzpatrick and Day 2004; Son et al. 2008; Mian et al. 2010; Muise et al. 2012). In addition, the short‐ and medium‐latency responses are reflected in GRF peaks at approximately 120–200 msec and 290–400 msec latency (c.f. Fig. 9), respectively, due partly to an electromechanical delay (Mian and Day 2009, 2014; Dakin et al. 2010; Mian et al. 2010; Horslen et al. 2014) and due partly to the twitch response time of muscle (Fig. 10 A, Appendix 2). These short‐ and medium‐latency responses in EMG and/or GRF data are well established since they were replicated in at least five different research institutions. According to Fitzpatrick et al. (1994) the short‐latency response can produce small segmental movements, but has no effect on the whole‐body sway response. It is generally assumed that the medium‐latency response is responsible for the GVS‐induced anodal whole‐body sway, however, the neurophysiological origin and function of the short‐latency response are still debated (Cathers et al. 2005; Mian et al. 2010). For example, while the short‐latency response occurs only in muscles required for balance, it is currently unclear whether the lower extremity short‐ and medium‐latency responses are independent or comprise a coordinated balance response (Fitzpatrick and Day 2004; Mian et al. 2010; Reynolds 2011).

In general, the relationships among vestibular‐evoked muscle activity, the resulting body movement, and the underlying physiological function remain unclear. Currently, there is insufficient knowledge of how muscle forces combine to produce movement in a nonrigid, multisegmental body. For example, the movement pattern related to the generation of the main anodal vestibular‐evoked sway response and to the manner in which it maps onto EMG and force plate data is insufficiently understood. Measurement of full‐body kinematics can integrate the effect of multiple measured and unmeasured muscle activations, can reveal patterns of movement, and allows us to parse the movement of the whole‐body centre of mass (CoM) into component parts (cervical, axial trunk, and appendices) so as to discriminate effects on cervical‐head stabilization (VCR), lower extremity balancing reflexes, and whole‐body balance. GRF measurements in isolation reveal acceleration of the CoM, but do not discriminate segmental movements. While a kinematic analysis of the head, trunk, and pelvis response to GVS has been made (Day et al. 1997), a full‐body kinematic analysis of the GVS response including the extremities has not been conducted to date. Such an analysis can be used (1) to differentiate the whole‐body centre of mass response (CoM) into component parts (cervical, axial trunk, appendicular short latency, and medium latency), (2) to unmask component responses which oppose and cancel within the whole‐body CoM response, (3) to assess the effect of fear of falling on each component, and finally (4) to discern their underlying mechanisms.

### Aims and approach

In this study we investigated how vestibular balance reflexes are influenced by fear of falling. It remains unknown whether, and to what extent, this psychological state modulates the vestibular reflex mechanisms involved in balance control. To challenge the balance system we used GVS to evoke substantial mediolateral sway both at ground level and at a height, a condition that is known to invoke fear of falling (Osler et al. 2013). We recorded full‐body kinematics to measure the balance response to GVS, in order to discriminate the VCR response from regulation of the CoM (i.e., the balancing response), and to gain insight into the neurokinematic progression of the balance response. We analyzed the collected kinematic data using statistical parametric mapping (SPM). SPM is a validated method of statistical analysis for time series data, which is now increasingly being used for kinematic time series where signals, typical from different segments, cannot be assumed to show peak effects at the same time (Pataky 2012; Robinson et al. 2014; Serrien et al. 2015). We focused on the short‐ and medium‐latency vestibular responses (0–400 msec). In our study we compared our full‐body kinematic data to known EMG and GRF responses as established in multiple laboratories. Our main research question was as follows: What is the effect of fear of falling on vestibular control of whole‐body balance? We divided this question into the following subquestions:

1. What is the kinematic response to GVS of axial and appendicular components, in the short‐ and medium‐latency time domain?
2. What is the effect of fear of falling on each of these components?
3. How do these components relate to each other and to the regulation of head stabilization and postural balance control?

## Methods

### Ethical approval

This study was approved by the local ethics committee of the Science & Engineering Faculty of Manchester Metropolitan University. Participants were naive to the precise purpose of the experiment and gave written informed consent prior to their participation. The study conformed to the standards set by the latest version of the Declaration of Helsinki.

### Participants

Sixteen young healthy adults with no known neurological, musculoskeletal, balance, or vestibular disorder were recruited as a sample of convenience. Ten men and six women were tested. The averaged participant characteristics were as follows: mean (standard deviation): age: 25.9 (5.1) years, height: 1.74 (0.1) m, weight: 69.5 (13.5) kg, BMI: 22.9 (3.5).

### Material

**Figure 1:**
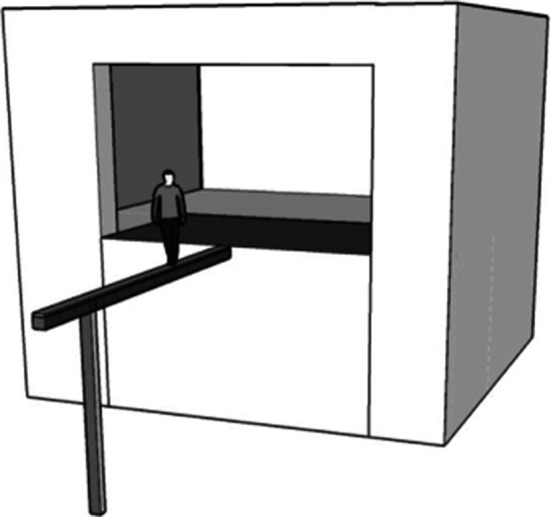
Narrow walkway at height.

Vestibular‐evoked balance responses were studied in two conditions. In one condition participants stood on a 22‐cm‐wide walkway placed on the laboratory floor. In the other condition, participants stood on a 22‐cm‐wide walkway elevated 3.85 m above ground level. The high walkway extended from a mezzanine into a larger neighboring room (Fig. 1). Access to the walkway was provided by sliding doors opening the laboratory wall (width 3.57 m). Stimulation and data acquisition devices were stationed on the mezzanine.

#### Safety system

In both the ground and height conditions participants wore a full‐body harness attached to a safety system to prevent a possible fall. The safety system consisted of an inertial reel and a dynamic rope system that was belayed by a certified assistant. Both were attached to a trolley‐mounted anchor point positioned directly above the participant to allow walking and standing without creating drag on the participant. This was the same safety system as used by Osler et al. (2013). As the system was attached to the back of the harness, the ropes ran behind the participant outside their visual field. Participants were fully informed of the safety system. However, during data collection, participants could neither see nor feel the safety ropes. Furthermore, they did not test the system prior to the experiment. Verbal, postexperiment debriefing confirmed that knowledge of the safety system provided little comfort to participants who generally reported the experience to be rather testing.

#### Data collection

Full‐body kinematics was collected by means of a 3D motion capture system operating at a sample frequency of 100 Hz using 52 retro‐reflective passive markers and nine infrared cameras (Vicon Motion Systems Ltd., Oxford, UK). The marker placement was as follows: five on the head (frontal bone, two on left, and two on right zygomatic bone), two on sternum, upper back at C7, lower abdomen, five on pelvis (ASIS, PSIS, and sacrum), upper lateral thigh (iliotibial band), five per knee (femoral and tibial condyles, and tibial tuberosity), lower lateral shanks, medial and lateral ankles, two per foot (heel and base of the third metatarsal), shoulders (acromion), upper arms (deltoid insertion), medial and lateral elbows, lateral lower arms (ulna shaft), two per wrist (radial and ulnar styloid process), one per hand (second metacarpal head).

Furthermore, skin conductance was recorded during all trials and served as a measure of physiological arousal. Skin conductance was measured using two self‐adhesive gel electrodes that were placed on the palmar surface of the distal phalanges of the first and third fingers. The electrodes were connected to a GSR Amplifier (ADinstruments Ltd., model ML116, Dunedin, New Zealand). Kinematics and skin conductance data were collected and synchronized using Vicon Nexus software (1.8.5.61009 h, Vicon Motion Systems Ltd., Oxford, UK). GVS impulses with a current of 1 mA and 2 sec duration were delivered using carbon rubber electrodes (46 by 37 mm) placed in a binaural bipolar configuration similar to the method of Osler et al. (2013). This type of stimulus has shown to evoke significant body sway responses (Day et al. 2010; Osler et al. 2013).

To assess participant's state of fear, the State‐Trait Anxiety Index (STAI) (Rossi and Pourtois 2012) was used. From the STAI questionnaire only the state anxiety index was used. Moreover, participants were asked to verbally rate their fear of falling on a 1–10 Likert‐scale anxiety thermometer at several instances of the experiment. The anxiety thermometer has been shown to have fair validity and reproducibility (Houtman and Bakker 1989). In a more recent study a one‐question 5‐point Likert anxiety scale was found to be suitable for anxiety measurement (BinDhim et al. 2013).

### Procedure

In a repeated measures design participants were tested during the same series of trials in the high and ground walkway conditions in counterbalanced order. Participants were instructed to stand still but relaxed 1.5 m out on the walkway with their head facing forward and the feet directed along the anterior‐posterior axis of the walkway (Fig. 1). To maximize lateral sway and rule out effects of vision, participants stood with their feet together and eyes closed. After 10 familiarizing GVS stimuli, 30 GVS impulses (15 anode left, 15 anode right, randomly ordered) were applied. It is important to note that the direction of body sway evoked by the stimulus was always toward either the right or the left edge of the walkway, depending on GVS polarity (anode left or right). Participants were permitted to open their eyes after each block of 10 trials. These trials were repeated, meaning that all participants completed three blocks of 10 trials in both the height and the ground condition. Data acquisition for each trial began 3 sec prior to and ended 6 sec following GVS onset. After each sixth trial in the first block, each eight trial in the second block, and each third trial in the third block of trials participants were asked to verbally rate their level of fear of falling for the anxiety thermometer.

### Data processing

Baseline skin conductance was calculated as the mean skin conductance level over 2 sec of quiet standing at ground level. Pre‐ and post‐GVS onset skin conductance levels were calculated by averaging skin conductance between 3 and 0.5 sec before GVS onset, and between 0 and 6 sec after GVS onset, respectively. Skin conductance signals were normalized by subtracting the baseline signal and dividing by the standard deviation of the pre‐GVS values in the ground condition.

**Figure 2:**
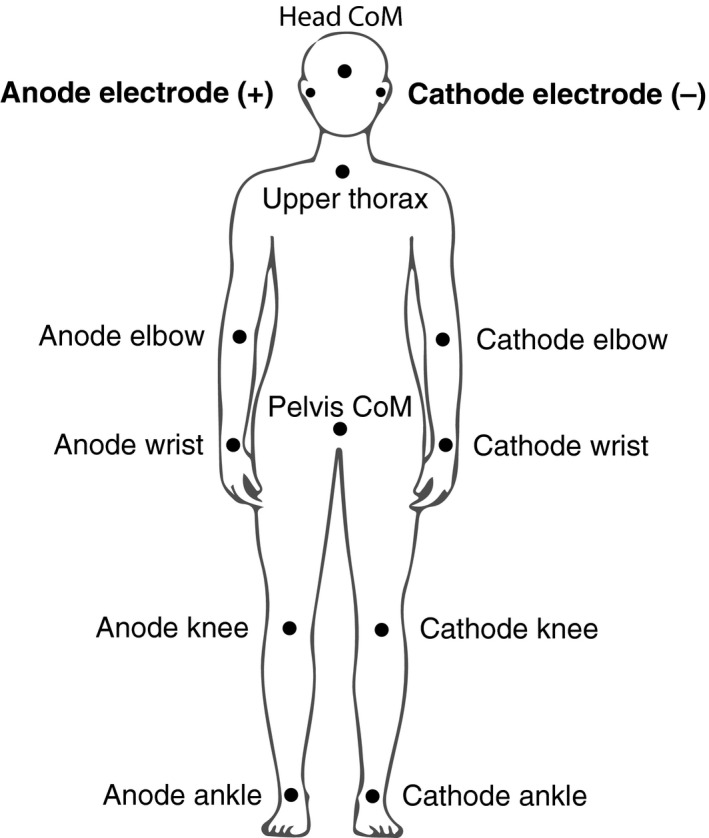
Body nodes based on GVS electrode configuration. We focused on mediolateral linear acceleration of the indicated body nodes. These nodes were analyzed based on the anode–cathode configuration as the GVS polarity changed between trials.

Using Visual 3D (v5.02.07, C‐Motion Inc., Germantown) mediolateral displacement of the following body nodes was calculated: whole‐body CoM, head CoM, upper thorax (superior end of thorax segment), pelvis CoM, and the elbows, wrists, knees, and ankles. These locations are collectively referred to as nodes. In addition, foot‐in‐space and head‐in‐space segment angles as well as ankle, knee, hip, lower back, neck, shoulder, elbow, and wrist joint angles in the frontal plane were calculated. A GVS stimulus causes increased mediolateral body sway to the side on which the anode electrode is placed on the head. For half of the GVS trials the anode of the GVS electrodes was on the right side and for the other half of the trials it was on the left side. Therefore, instead of analyzing right and left body nodes and angle variables on their own (e.g., right or left knee), these segments were analyzed and named based on the anode–cathode configuration, for example, ‘anode knee’ refers to the knee on the anode side of the body (Fig. 2).

For each positional and angular variable, the value at GVS onset of a trial was subtracted from all values of the time series of the trial in question. Furthermore, the sign was corrected based on anode electrode location. Analysis of published data shows that the frequency bandwidth of the short‐ and medium‐latency GRF GVS responses averaged over multiple trials and participants does not exceed 3 Hz (Marsden et al. 2005; Mian and Day 2014). Therefore, we filtered our kinematic data using a 6 Hz low pass Butterworth filter and differentiated twice using a third‐order Savitsky‐Golay filter with a temporal window of 170 msec (Press et al. 1999). As we were interested in the vestibular reflex response we analyzed node acceleration and angle acceleration data in the time domain between 0.2 sec before and 0.7 sec after GVS onset.

### Statistics

#### Questionnaire and skin conductance data

Student's paired *t*‐tests were used to test whether STAI state, anxiety thermometer ,and skin conductance were increased at height compared to ground. Lastly, correlations between all combinations of skin conductance, anxiety thermometer scores, and STAI state scores were calculated using Spearman's rho. The statistics toolbox in Matlab was used for statistical testing.

#### Kinematics: SPM

To answer our research question all linear and angular acceleration time samples within the first 400 msec after GVS onset were of interest. We therefore used a validated method (SPM) to test at what instances the signals were statistically different from zero and when they were different between conditions. All SPM analyses were implemented using the open‐source toolbox SPM‐1D (v.M0.1, Todd Pataky 2014, www.spm1d.org) in Matlab R2014a. SPM is now increasingly used in the analysis of kinematic time series (Pataky 2012; Robinson et al. 2014; Serrien et al. 2015), as it overcomes the limitation of confining statistical testing to scalar data (e.g., a single instant in time). SPM allows time dependence of effects to be incorporated directly in statistical testing by using the whole time series as the unit of observation.

In this study SPM statistics were calculated for the averaged trials per participant for each condition. Relevant to question 1 above, a SPM two‐tailed one‐sample *t*‐test was used separately for the ground and height condition data to test if linear and angular acceleration of previously mentioned body nodes, joints, and segments was different from zero (*α *= 0.05). Additionally, relevant to research question 2, a SPM two‐tailed paired samples *t*‐test (Robinson et al. 2014) was used for a ground versus height comparison of the same dependent variables. The scalar output statistic, SPM[t], was calculated separately at each individual time sample. To test the null hypothesis the critical threshold was calculated as the value at which only *α* % (5%) of the analyzed trajectories would be expected to traverse. This threshold of significance is based upon estimates of trajectory smoothness and Random Field Theory expectations (Adler and Taylor 2007). Conceptually, a SPM *t*‐test is similar to the calculation and interpretation of a scalar *t*‐test; if the SPM[t] trajectory crosses the critical threshold at any time sample, the null hypothesis is rejected. However, a SPM *t*‐test avoids the false positives of multiple scalar *t*‐tests and avoids the false negatives of scalar *t*‐tests with Bonferroni correction (Adler and Taylor 2007). Typically, due to interdependence of neighboring points, multiple adjacent points of the SPM[t] curve often exceed the critical threshold. We therefore call these “supra‐threshold clusters”. SPM then calculates cluster‐specific *P*‐values which indicate the probability with which suprathreshold clusters could have been produced based on the null hypothesis (Adler and Taylor 2007).

## Results

### Questionnaires and skin conductance confirm increased fear of falling at height

STAI, anxiety thermometer and skin conductance data showed that participants had a higher level of fear of falling and physiological arousal in the high walkway condition than in the ground walkway condition (Table 1). Skin conductance was increased significantly in the height condition both pre‐ (*t *=* *−2.709, *df* = 15, *P *=* *0.016) and post‐ (*t *=* *−2.743, *df* = 15, *P *=* *0.015) GVS onset. In the height condition, the STAI state scores were positively correlated with skin conductance scores (*n *=* *15, *ρ* = 0.506, *P *<* *0.05). For one participant skin conductance was not recorded due to technical malfunction.

**Table 1:** STAI, anxiety thermometer, and skin conductance scores

	STAI State	Anxiety thermometer	Skin conductance	
			Pre‐GVS	Post‐GVS
Ground	27.4 (5.7)	2.0 (1.1)	−0.53 (1.08)	−0.45 (1.19)
Height	34.8 (9.3)	4.7 (3.2)	3.91 (6.11)	4.02 (6.08)

The data are presented as mean (SD). State anxiety scores (STAI) can range between 20 and 80. Anxiety thermometer scores can range between 1 and 10. Skin conductance values are normalized to values of baseline standing.

### Kinematic analysis of vestibular responses to GVS

#### Representative response of the whole‐body CoM

**Figure 3:**
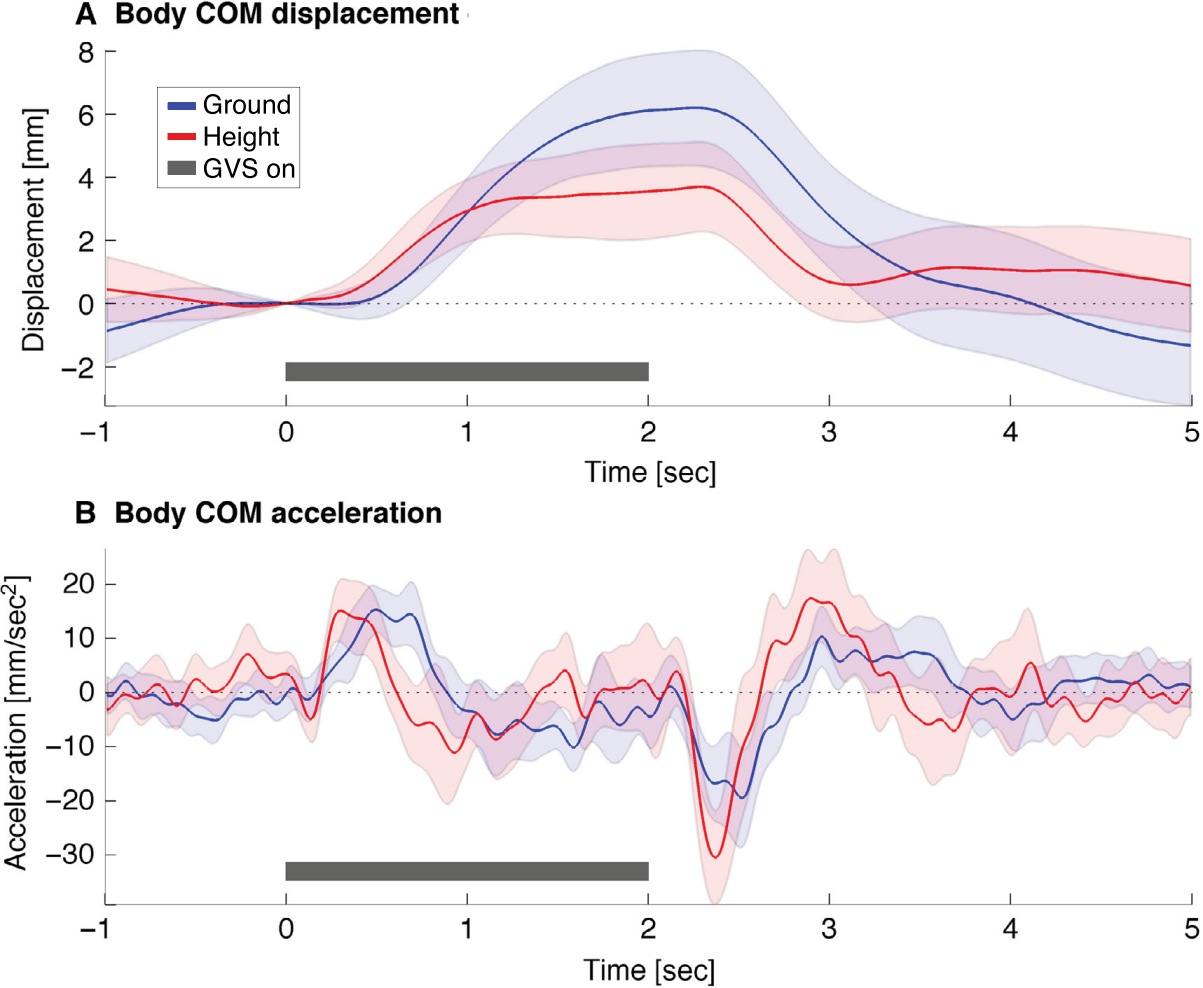
Effect of height on body CoM response to GVS of representative participant. The mediolateral body CoM displacement (A) and acceleration (B) of one participant are shown. GVS onset occurs at 0 sec and ends at 2 sec. Lines represent (individual) condition means and shaded areas represent 95% confidence intervals of the trials. The black bar shows the time at which GVS was on. For each trial, CoM displacement was scaled to *t* = 0, that is, GVS onset.

Standing at height has a modest effect on the early sway response (before ~400 msec), and a clear effect on the late GVS body sway response after ~400 msec. Figure 3 shows an example of the whole‐body CoM mediolateral displacement and acceleration of a representative participant. At ~200 msec after GVS onset the whole‐body CoM started to accelerate toward the anode electrode in both the ground and height condition. However peak acceleration was reached at 490 msec at ground level and at 300 msec at height. The amplitudes of this anode‐directed (anodal) peak acceleration at ground and height were relatively similar. Whole‐body CoM started decelerating at 890 msec at ground level and at 610 msec at height. These changes resulted in a reduced maximum sway displacement at height compared to ground.

#### Whole‐body CoM group results

**Figure 4:**
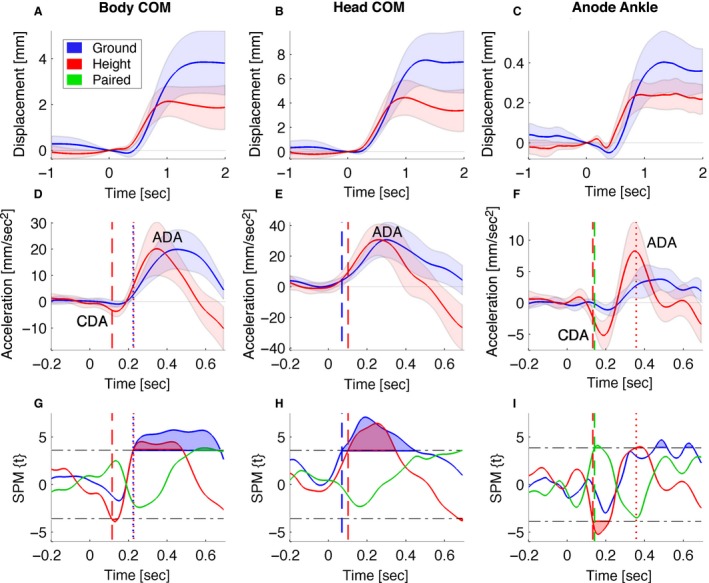
GVS effects and ground–height difference effects found on acceleration within 0.2 sec after GVS. The *left, middle, right* columns show movement of nodes for: whole‐body CoM, head CoM, and anode ankle, respectively. (A–C), Upper row, shows mediolateral position. (D–F), Middle row, shows mediolateral acceleration. Lines represent condition means and shaded areas represent 95% confidence intervals of the ground and height conditions. Anode‐ and cathode‐directed acceleration peaks are indicated by ADA and CDA, respectively. (G–I), Bottom row shows statistical parametric maps. Ground, height, and ground–height difference are in blue, green, and red, respectively. Lines represent statistical parametric mapping(t) time series of the separate one‐sample *t*‐tests for ground and height data and paired *t*‐tests for the ground–height difference. Horizontal dash‐dot lines are the thresholds of significance. Shaded areas are suprathreshold clusters that indicate the time domains with significant effects. GVS onset occurs at 0 s. Vertical dashed and dotted lines represent the onset of significant short‐ and medium‐latency acceleration, respectively. These vertical dashed and dotted lines are shown for significant effects in the ground and height conditions, as well as for the ground–height difference.

GVS‐evoked whole‐body CoM sway toward the anode (positive) was conventional in that it plateaued at ~1 sec, and was preceded by a small cathode‐directed (cathodal) peak (negative) at ~250 msec (Fig. 4A). The whole‐body CoM showed a small initial cathodal acceleration and a main anodal acceleration of ~ 20 mm sec^−2^. The timing of cathodal and anodal acceleration responses showing peaks at ~130 msec and at ~400 msec (Fig. 4D) was comparable to short‐ and medium‐latency vestibular reflex responses found previously in GRF data (Fig. 9).

At height, cathodal acceleration was significantly different from zero at 120–140 msec (*P *=* *0.027, *t* = −3.67, mean ± 95% confidence interval value at peak −3.668 ± 2.0 mm sec^−2^) followed by significant anodal acceleration at 230–470 msec (*P *<* *0.001) (Fig. 4D, G). In the ground condition no significant cathodal acceleration was found, however, anodal acceleration was significant at 230–670 msec (*P *<* *0.001) (Fig. 4G). At 550–650 msec the ground–height difference was significant (*P *<* *0.001) for body CoM acceleration (Fig. 4G). The ground––height time difference between anodal acceleration peaks was 110 msec and the body CoM sway terminated more promptly by ~300 msec at height (Fig. 4A).

#### Head CoM & upper thorax nodes

**Figure 5:**
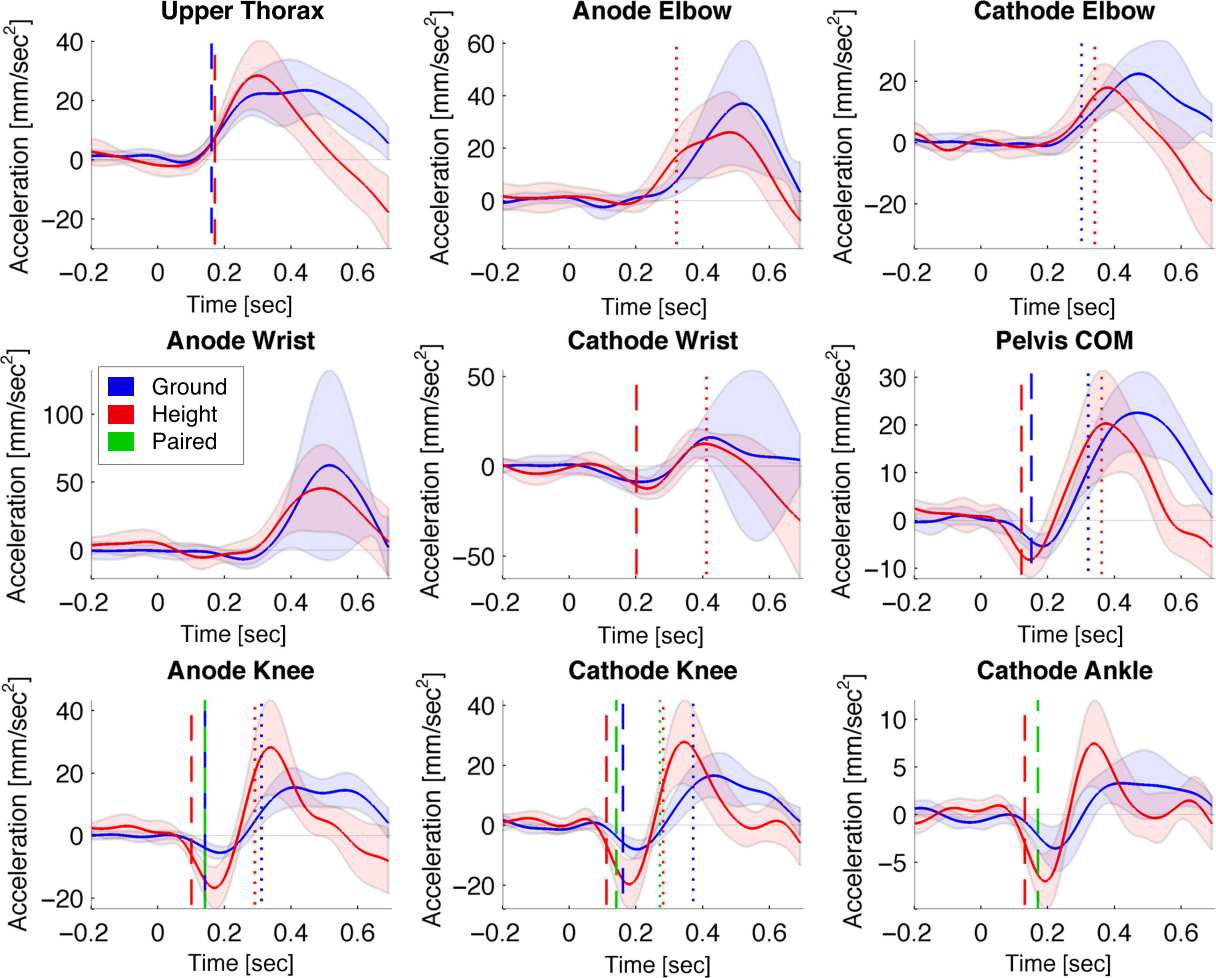
Cathodal acceleration around ~0.2 sec in pelvis and lower extremities only. Data are shown of all nodes that are not included in Figure 4. Nodes are ordered from superior to inferior. Lines represent condition means and shaded areas represent 95% confidence intervals of the ground and height conditions. Positive values are mediolateral anodal acceleration and negative values are mediolateral cathodal acceleration. Vertical dashed and dotted lines represent the onset of significant short‐ and medium‐latency acceleration, respectively. These vertical dashed and dotted lines are shown for significant effects in the ground and height conditions, as well as for the ground–height difference.

Following GVS, the head node swayed consistently to the anode before plateauing at ~1 sec (Fig. 4B). Initial acceleration of the head CoM and upper thorax node was anodal (Fig. 4E). Head CoM acceleration was significant from 70 msec (*P *<* *0.001, Fig. 4H) and upper thorax acceleration was significant from 160 msec (Fig. 5).

The anodal acceleration of the head and upper thorax nodes was unaffected by height. No significant ground–height difference was found for head CoM or upper thorax within the first 0.4 sec (Fig. 4H). This lack of a significant difference between height and ground replicates the head and trunk kinematics collected by Osler et al. (2013).

#### Response of the lower extremities: pelvis, knee, and ankle nodes

Initial cathodal acceleration was observed in the pelvis and lower limbs. This response occurred at short latency and was followed by anodal acceleration at medium‐latency (Figs 3 and 4). For both knees, both ankles, and pelvis, cathodal acceleration was significant from 100 to 150 msec (Figs 4F and 5). These short‐latency cathodal acceleration clusters were followed by significant medium‐latency anodal acceleration clusters (pelvis and knees), which started between 270 and 370 msec (Fig. 5).

The effect of height was an increase in the magnitude of the initial cathodal acceleration in the lower limbs. Inspection of Figures 4F and 5 shows that this increase was largest for the knee and ankle nodes, as confirmed by the significant ground–height difference in the initial cathodal acceleration. Cathodal acceleration was also observed earlier at height (Figs. 4F and 5).

#### Response of the upper limbs: elbow and wrist nodes

The upper limbs showed a clear anodal acceleration at medium latency with a notable absence of a response at short‐latency timescales (Fig. 5). Only the cathode wrist showed a significant cathodal response at short latency. The amplitude was similar to the pelvis CoM; therefore, the pelvis acceleration could have been transferred mechanically to the cathode wrist. No significant difference between ground and height was found (Fig. 5).

#### Summary of whole‐body GVS response revealed by node movements

**Figure 6:**
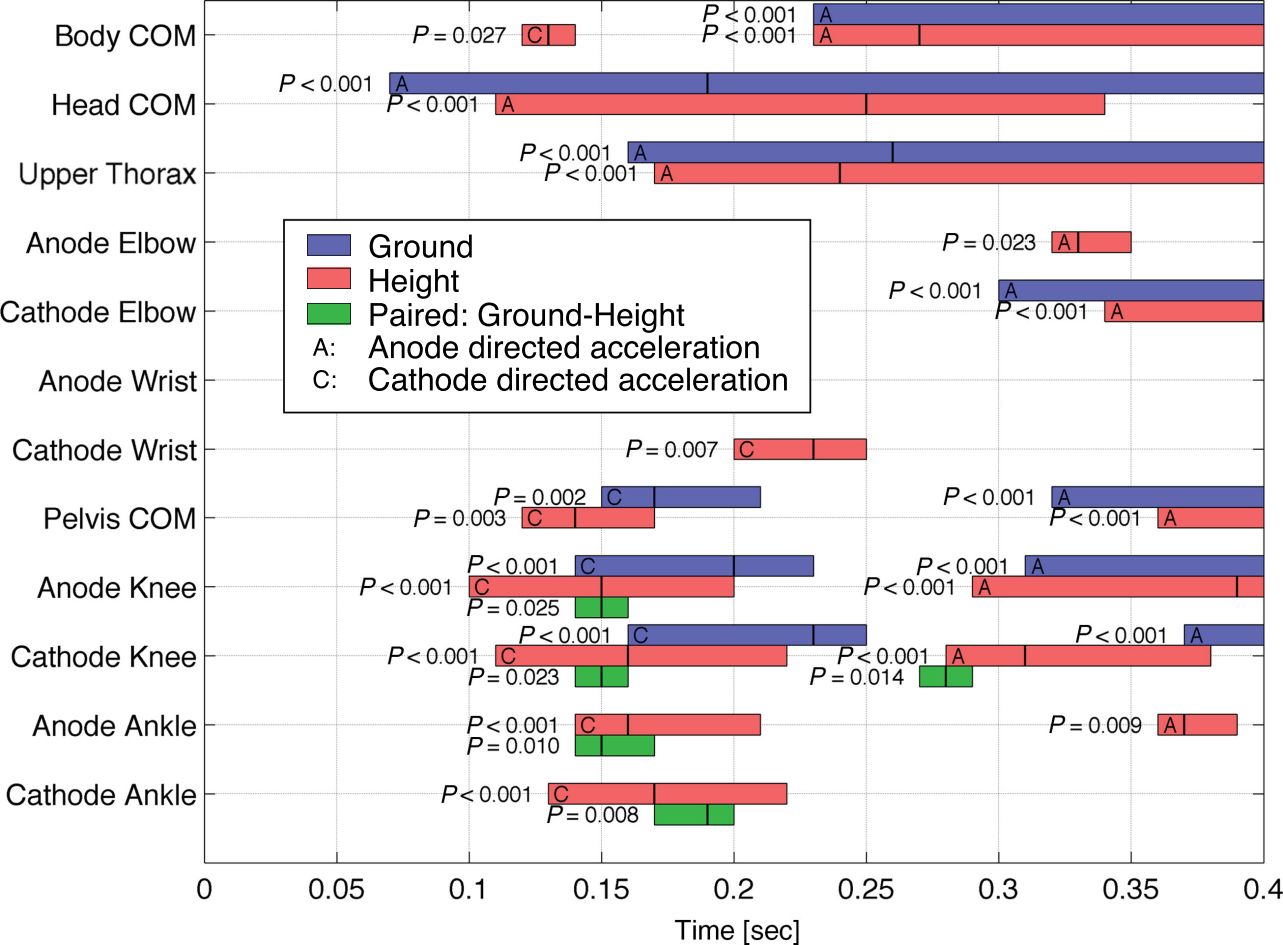
Body node acceleration: Significant time domains at ground vs. height. The bars show significant time domains of the SPM one‐sample *t*‐tests for ground and height, and the SPM paired *t*‐tests on the ground–height difference. Vertical lines within each suprathreshold cluster bar indicate the time of maximum significance. The *P*‐value of each cluster is shown left of each bar. Significant short‐latency ground–height differences within 0.14–0.2 sec were found in acceleration of lower extremity nodes only. A significant medium‐latency ground–height difference was found for cathode knee only from 0.27 to 0.29 sec.

Figure 6 provides a sequential overview of the GVS acceleration response and the effect of height for all body nodes. The whole‐body response (Body COM) integrates all component parts. The GVS response comprises an early anodal acceleration of the head and upper thorax, an overlapping, oppositely directed, short‐latency cathodal acceleration of the pelvis and lower limbs and a subsequent medium‐latency anodal acceleration of the whole‐body CoM resulting in sustained anodal sway of the whole body.

Cathodal acceleration had a short‐latency origin, was restricted to the pelvis and lower limbs, and showed a response pattern with a strongest, earliest effect at the knee. We describe this cathodal acceleration pattern, strongest at the knee, as “cathodal buckling”. The effect of height‐induced fear of falling on vestibular reflexes was significant only in acceleration of lower extremity nodes.

**Figure 7:**
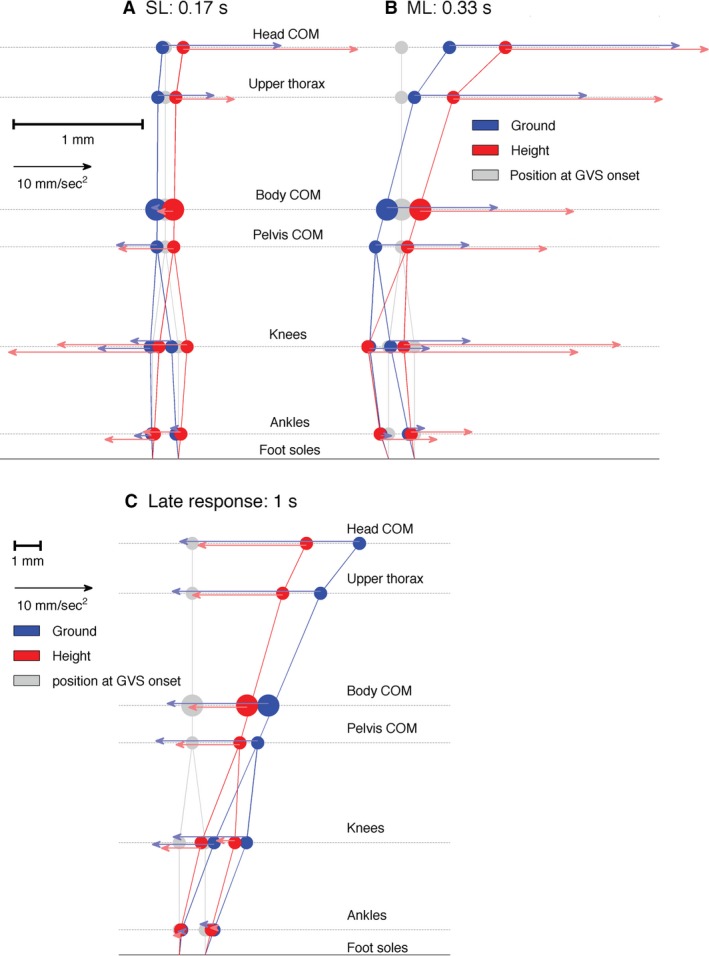
Nodes at different times after GVS onset. Dots and stick figures show mediolateral displacement of the head, trunk, and lower extremity body nodes with respect to the position at GVS onset. This displacement is shown for three different points in time. For each stick figure the left side represents the cathode side and the right side represents the anode side. Arrows represent mediolateral acceleration. At the three time points, short‐latency (A), medium‐latency (B), and late (C) acceleration responses are shown. Mediolateral displacement and acceleration scales are shown in the legend. Note that the node position scale for the lower stick figures (C) is 5 times smaller than the scale for the top stick figures (A–B). Internode distances are not scaled.

Figure 7 shows the mean displacement and acceleration at key time points. A video of the GVS response showing movement of stick figures comparable to Figure 7 can be found in Supplementary Material and is described in Appendix 1. Cathodal buckling of the lower extremity is evident initially (Fig. 7A) from the pattern of cathodal acceleration vectors, which are largest, at the knee, and later (Fig. 7B) from the node displacement (knee buckling). At 170 msec, comparable with the GRF short‐latency response, the cathodal acceleration and its increased magnitude at height was evident at the ankle, knee, and pelvis nodes. At 330 msec, comparable with the GRF medium‐latency response, acceleration and displacement of the whole body toward the anode was associated with cathodal buckling of the lower limbs centered at the knee, and this effect was also increased at height.

#### Joint and segment angle acceleration

Node movements result from a combination of joint rotations. For example, head node movement summarizes the cumulative rotation of joints from the ankles to the neck. The following results remove ambiguity regarding the source of the head and trunk node accelerations.

**Figure 8:**
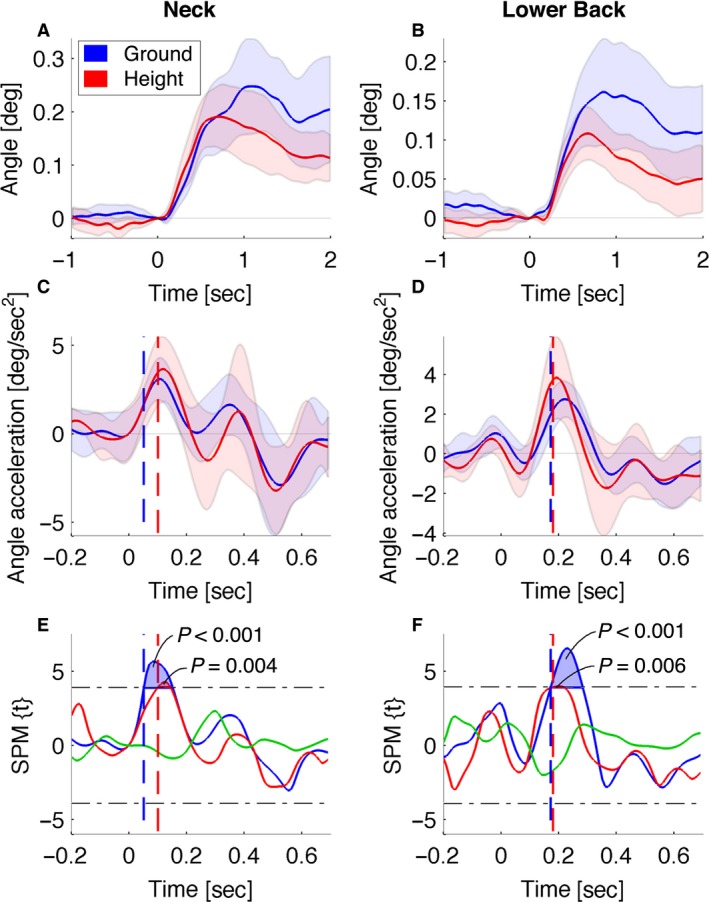
GVS effects in both conditions for angle accelerations, no ground–height difference effects. The *left and right* columns of graphs represent neck lateral flexion and lower back lateral flexion, respectively. Positive values represent anode flexion, that is, folding together of the proximal and distal segments of the joint toward the lateral side on which the anode electrode is placed. (A & B), The first row, shows lateral flexion angles. (C & D), the second row, shows angle acceleration. Lines represent condition means, and shaded areas represent 95% confidence intervals of the conditions (ground and height). Positive values are lateral flexion toward anode and negative values are lateral flexion toward cathode. (E & F), The bottom row shows statistical parametric maps. Lines represent SPM(*t*) time series of the separate one‐sample *t*‐tests for ground and height data and paired *t*‐tests for the ground–height difference. Horizontal dash‐dot lines are the thresholds of significance and shaded areas are suprathreshold clusters that indicate the time domains with significant effects. GVS onset occurs at 0 sec. Vertical dashed and dotted lines represent the onset of significant short‐ and medium‐latency acceleration, respectively. These vertical dashed and dotted lines are shown for significant effects in the ground and height conditions. No significant ground–height difference effect was found in any of the measured angles.

Initial linear anodal acceleration of the head (50–100 msec) and upper thorax nodes (100–150 msec) (Fig. 6) were confirmed as arising from joint rotations at the neck and subsequently the lower back (Fig. 8A and B). In both conditions the VCR was faster than the vestibular reflex in any of the other joints. Height had no significant effect on the magnitude of these axial reflexes (Fig. 8E and F), which were remarkably consistent at ground and height (Fig. 8A and B).

## Discussion

The goal of this study was to investigate the effects of fear of falling on vestibular control of whole‐body balance with the following subquestions:

1. What is the kinematic response to GVS of axial and appendicular components, in the short‐ and medium‐latency time domain?
2. What is the effect of fear of falling on each of these components?
3. How do these components relate to each other and to the regulation of head stabilization and postural balance control?

### Kinematic analysis shows both a unidirectional anodal, and a bidirectional (cathodal‐anodal) response to GVS

**Figure 9:**
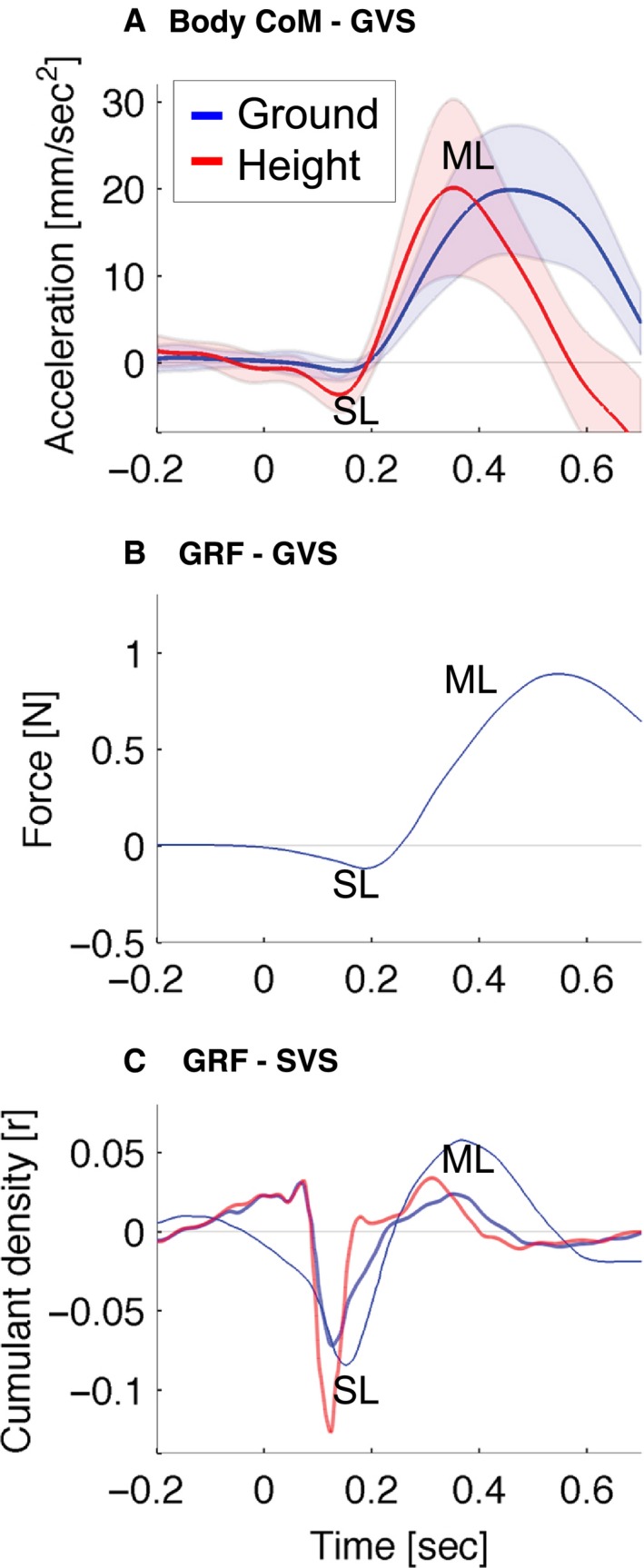
Short‐ (SL) and medium‐latency (ML) responses in different publications. (A) Mediolateral acceleration of body CoM from this study at ground and height is shown. Acceleration toward the anode GVS electrode (ADA) is positive and cathode‐directed acceleration (CDA) is negative. A 1 mA GVS stimulation starts at 0 sec with 2 sec in duration. (B) This graph is redrawn from Marsden et al. (2005). A 1 mA GVS of 3 sec duration starts at 0 sec and the shear GRF is plotted. GRF toward anode is positive and toward cathode is negative. Participants stood at ground level. (C) SVS‐GRF coupling (cumulant density) is shown as a function of the SVS‐GRF time lag. GRF‐SVS (2–25 Hz) cumulant density of participants standing at low and at high altitude is shown by the thick lines. These data are redrawn from Horslen et al. (2014) so that positive values indicate coupling of vestibular stimulation (SVS) with shear GRF toward anode and negative values indicate coupling of SVS with shear GRF toward cathode. The thin line shows GRF‐SVS (1–20 Hz) cumulant density data at ground level redrawn from Mian et al. (2010). The short‐ and medium‐latency (SL and ML) responses follow a pattern that is comparable to the short‐ (CDA) and medium‐latency (ADA) responses found in the body CoM and lower body nodes with GVS in this study. SVS, stochastic vestibular stimulation.

**Figure 10:**
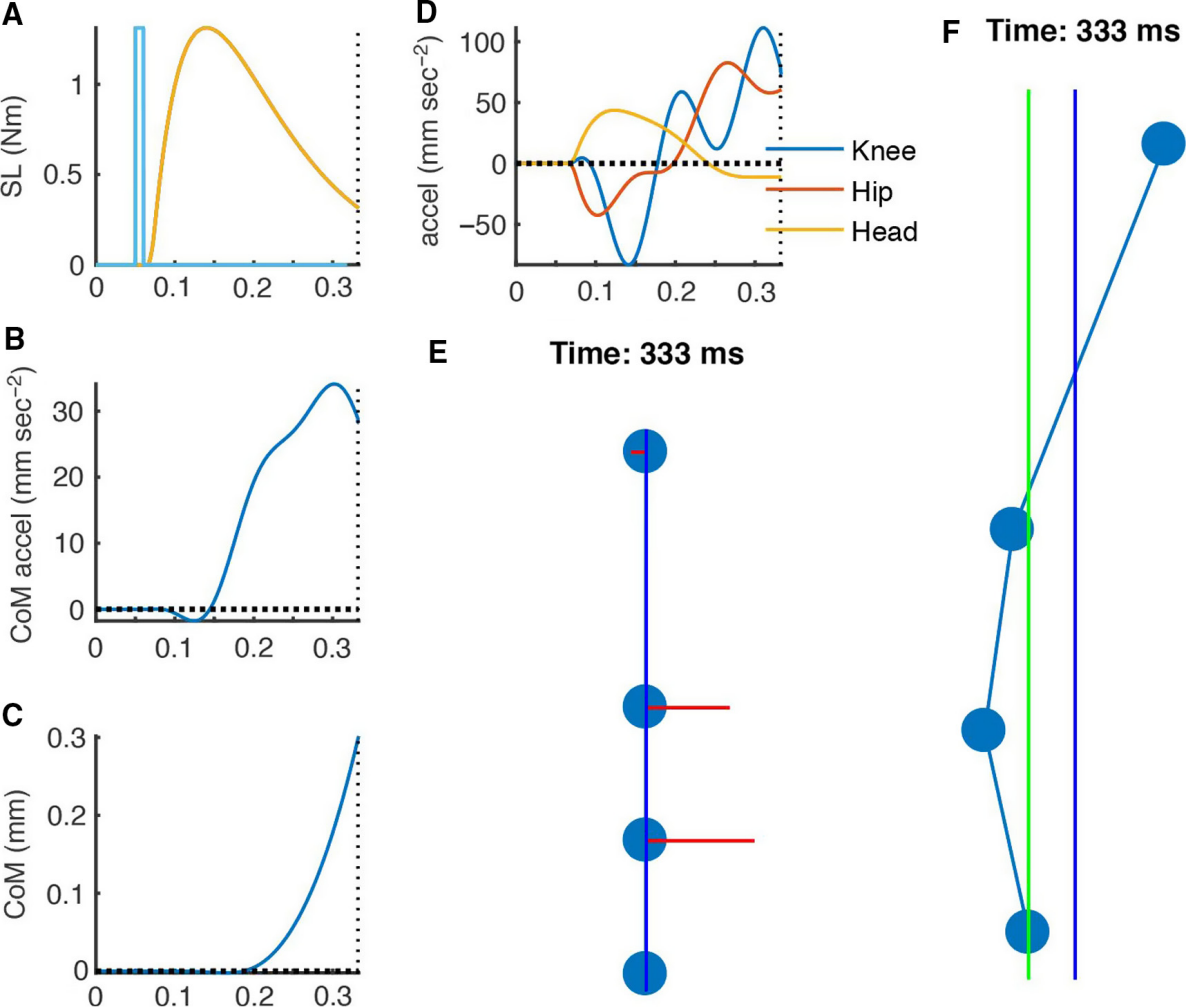
Figure 10 and simulation videos all simulation videos contain the same panels as described for Figure 10. For all panels motion toward the anode is positive and frames indicate time in msec from the start of the electrical stimulus. Figure 10 shows the data of frame 333 from the video of simulation 4. (A), EMG timing (no scale) is shown by the blue line. Moment (Nm) applied to each joint in the simulation is shown by the yellow line. After a 15 msec delay, generation of joint moment begins with a peak at approximately 130 msec. (B) Acceleration of whole‐body centre of mass (CoM, mm sec^−2^) (C), Sway of CoM (mm) (D), Acceleration of the knee, hip, and head nodes (blue, red, yellow, mm sec^−2^). (E) Position of the ground contact, knee, hip, and head nodes at 333 msec after the start of the electrical stimulus. Horizontal and vertical axes show equal scale. Red lines show node acceleration, direction, and relative magnitude. (F) Position of the ground contact, knee, hip, and head nodes at 333 msec after the start of the electrical stimulus. Maximum horizontal scale limits are set at 1 mm to magnify movement of the nodes. Centre of mass projection is shown by the vertical blue line and projection of the ankle joint by the vertical green line.

Our results show a unidirectional, anodal acceleration of the head CoM and upper thorax in response to GVS (Figs. 6 and 7). This anodal acceleration is consistent with previous findings (Osler et al. 2013; Forbes et al. 2014). Our novel findings in the body CoM, pelvis, and lower limbs showed a bidirectional pattern of cathodal acceleration (cathodal buckling of the lower extremity) followed by anodal acceleration of the whole body (Figs. 6 and 7). This biphasic pattern is consistent with the well‐established short‐ and medium‐latency GRF and EMG responses to vestibular stimulation (Britton et al. 1993; Fitzpatrick et al. 1994; Ali et al. 2003; Fitzpatrick and Day 2004; Son et al. 2008; Mian and Day 2009, 2014; Dakin et al. 2010; Mian et al. 2010; Muise et al. 2012; Horslen et al. 2014). This pattern is also consistent with a small cathodal sway preceding the larger anodal sway of the pelvis shown previously by Cathers et al. (2005) in their Figure 2. For reference, Figure 9 shows published GRF records of the short‐ and medium‐latency responses and confirms that our acceleration data are showing short‐ and medium‐latency responses within the lower extremity. For reference also, Figure 10A, (Appendix 2) illustrates the timing of short‐latency muscle force production from short‐latency EMG responses.

### Cathodal buckling of the lower extremity is a mechanical consequence of generating anodal sway of the whole body

The main GVS‐evoked sway response of the whole body was toward the anode. The GVS‐evoked generation of momentum of the whole‐body centre of mass relative to the ground, toward the anode, requires generation of a moment of force acting on the whole‐body centre of mass relative to the ground. To ensure mechanical transmission between ground contact and whole‐body centre of mass, sufficient moment must be generated between the whole‐body centre of mass location and the ground. In our experimental standing configuration, this GVS‐evoked moment between ground and centre of mass can arise only from muscles of the lower limb. As the lower limb is a nonrigid kinetic chain, this generation of a lateral flexion moment across the whole lower limb must evoke a cathodal buckling movement within the lower limb (c.f. Appendix 2 “Link model simulation of response generated at short‐latency (SL)” and Simulations [Link], [Link], [Link], [Link] in Supplementary Material). The exact profile of the cathodal “buckling” of the lower extremity depends upon the acceleration of individual linked segments and is determined by the distribution of inertia, joint stiffness, and distance from the more inert ends of the chain (ground, trunk). We observed acceleration highest at the knee (Fig. 7). While the location of highest acceleration is not important, the presence of a cathodal buckling pattern within the lower extremity is a mechanical signature of generating anodal sway of the whole body.

Note that acceleration of the head generated axially at the neck (e.g., the VCR) produces an anodal buckling of the body below the neck (Simulation 5, Appendix 2) and thus cannot explain the pattern of cathodal acceleration in the lower extremity. Likewise acceleration of the trunk generated axially at the lower lumber region produces anodal acceleration and buckling of the body below. Our results showed no active acceleration of the upper limbs until medium latency (Fig. 6). The short‐latency reflex is the only physiological reflex available to explain the observed cathodal acceleration of the lower extremity at short latency.

### Does the short‐latency response contribute to balance control?

The appearance of a cathodal buckling pattern of acceleration at short‐latency indicates a purpose at short latency to generate anodal sway of the whole‐body centre of mass. This observation of short‐latency cathodal buckling links the short‐ and medium‐latency responses to a common purpose, which would be the regulation of balance (Day et al. 1997). Our results also indicated that fear of falling accentuates the biphasic response both at short and medium latency (Figs. 6 and 7). This common modulation by fear of falling adds weight to a hypothesis that short‐ and medium‐latency responses are coordinated to a common purpose of balance regulation.

Within the literature diverse views are explored concerning the sensory origin and function of the short‐latency response, as well as its coupling or independence with the medium‐latency response (Fitzpatrick and Day 2004; Cathers et al. 2005; Mian et al. 2010; Reynolds 2011; Horslen et al. 2014). We propose the general hypothesis that the short‐ and medium‐latency responses comprise a coordinated balance response. This hypothesis arose unexpectedly following reflection upon the results of our kinematic analysis. Our experiment has limitations, which preclude the general testing of this hypothesis. For example, we studied one stimulus current only, and one postural configuration only. Based on the literature we would predict that the strength of response at short and medium latency would differ for a range of currents (Fitzpatrick et al. 1994; Fitzpatrick and Day 2004), which does not test the pattern of response. A stronger test of the hypothesis would be to alter the configuration of the participant to establish whether or not the response at short latency contributes to the sway observed at medium latency. Extant published data in which posture was altered support the general hypothesis. For example, Reynolds (2011) altered head pitch systematically and showed craniocentric modulation of ground reaction torque, including reversal of sign, at short and medium latency. Horslen et al. (2014) altered head yaw and showed craniocentric modulation of ground reaction force at short and medium latency. Forbes et al. (2016) altered head yaw systematically and showed craniocentric modulation of muscle activities at short and medium latency. Mian and Day (2014) altered stance width and head orientation and showed balance relevant modulation of response at short and medium latency. Nevertheless, Mian et al. (2010) might be cited as evidence indicating that short‐ and medium‐latency responses are uncoupled. However, we suggest their data support the hypothesis of a coordinated response, if one considers that within their head down condition the craniocentric axis of rotation passes in front of the body rather than through the whole‐body centre of mass. Following our results of cathodal buckling and that of the literature, we predict that the short‐ and medium‐latency responses comprise a coordinated regulation of balance. We predict that for different body configurations, the expression in EMG, GRF, and movement at short and medium latency will reflect the coordinated pattern needed to regulate whole‐body CoM in that configuration.

### Fear of falling influences vestibular balancing responses, but not the VCR

Vestibular sensation enables closed‐loop feedback control of the centre of mass horizontal position (balance) and head orientation (VCR) (Day et al. 1997; Forbes et al. 2014, 2016). Whether and how fear of falling influences these physiological control systems is currently debated (Horslen et al. 2015a,b; Reynolds et al. 2015a,b). Our analysis reports time‐series movement data low pass filtered at 6 Hz. This analysis bandwidth was used on account of the frequency content of the self‐generated movement (0–3 Hz)(Grossman et al. 1988) rather than our measurement system which had an analysis bandwidth of 50 Hz and sensitivity to movements <0.1 mm and 1 mm sec^−2^ (Figure 4 CFI). Within this bandwidth (0–6 Hz), and for the stimulus current tested (1 mA), our results show that neck‐generated head movement (VCR) was highly consistent between repetitions and between conditions (Fig. 8A). The early acceleration response of the head and upper thorax was unaffected by fear of falling. Only for the lower limbs was early GVS‐induced acceleration increased significantly by fear of falling.

### Evaluation of study limitations

Statistical significance was demonstrated in movements of remarkably small amplitude (Fig. 4CFI). This confirms the sensitivity of our experiment and underscores the extent to which early acceleration of the head and upper thorax arising from angular acceleration of the neck and lower back were not influenced by fear. With the head forward configuration (as used in our study) the coherence bandwidth of electrically evoked force plate responses linked to head orientation lies at 2–3 Hz (Dakin et al. 2007; Reynolds 2011). This bandwidth includes those responses modulated at short and medium latency by head pitch (Reynolds 2011). Coherent responses at higher frequency (7–8 Hz) have been interpreted as reflecting general mechanical transmission, unmodulated by head orientation (Reynolds 2011). Higher bandwidth coherences beyond 6 Hz are typically seen in the sagittal plane with head turned (e.g., 28). Compared with other sensorimotor feedback loops, the VCR bandwidth is particularly high, showing coherence in the fastest muscles up to 70 Hz between muscle activity and electrical stimulus (Forbes et al. 2014). However, the bandwidth of head movement is an order of magnitude lower, and lower still for self‐generated movement rather than movement generated by external impact (Viviani and Berthoz 1975; Grossman et al. 1988; Pozzo et al. 1990). Effects of fear in VCR muscle output beyond 6 Hz are unlikely to contribute power of consequence to head movement.

This study confirms that height‐induced fear of falling accentuates the short‐ and medium‐latency balance response and hence increases the response gain of vestibular‐evoked whole‐body stabilization. The functional effect of fear of falling is an earlier arrest of anodal sway, halving the distance moved by the whole‐body CoM toward the dangerous edge (Fig. 3).

Our findings are consistent with those of Horslen et al. (2014), who found an increased gain of the GRF‐SVS response at short and medium latency during postural threat. The present findings are also consistent with the seemingly opposing results of Osler et al. (2013), who found no effect of postural threat on early acceleration of the head and upper trunk. As they collected kinematics of head and trunk but not of the lower limbs, they concluded that fear of falling does not affect the vestibular balance reflex. Our study shows that fear of falling accentuates the vestibular balance response, as the gain of short‐ and medium‐latency responses found in lower limb kinematics was increased at height. Our study also shows that early CoM acceleration comprises the integration of anodal head acceleration and cathodal lower limb acceleration (Fig. 6). These opposing accelerations would mutually cancel and tend to reduce the early CoM acceleration signal, which was significant only at height. Hence a contribution of this study is a demonstration of limitations of CoM acceleration (and hence ground reaction force) to reveal GVS responses, and demonstrates the power of kinematic analysis to reveal opposing components of the GVS response.

### Axial head‐in‐space stabilization is task independent

Vestibular afferents are used in multiple feedback pathways for a variety of functional purposes. Regulating head‐in‐space orientation (neck stabilization) and regulating the whole‐body CoM to maintain balance can be distinguished as separate goals with different underlying mechanisms (Day et al. 1997; Forbes et al. 2015). These goals are related hierarchically in the sense that balance of the whole body depends upon integration of vestibular with proprioceptive information, which depends upon vestibular regulation of head orientation (VCR) (Forbes et al. 2014). Our results are consistent with others who see a distinction between vestibular mechanisms that govern axial and appendicular reflexes (Forbes et al. 2014, 2015).

Vestibulocollic neural pathways regulating head‐in‐space position mostly comprise three‐neuron arcs. They originate primarily from medial vestibular nuclei, and response latencies of these pathways are short (~8–10 msec) (Watson and Colebatch 1998; Forbes et al. 2014). Additionally, the VCR short‐latency response of the sternocleidomastoid (SCM) muscle response was unaltered by manipulation of vision, external support, stance width, and posture (Watson and Colebatch 1998; Welgampola and Colebatch 2001). Forbes et al. (2014) tested the effect of fixating the trunk and head position on the VCR with the idea that this fixation rendered the neck muscles irrelevant to head posture. The VCR was still present in the fixed condition and was therefore concluded to be task independent.

### Appendicular whole‐body stabilization is task dependent

The whole‐body sway response is task dependent and more flexible than the VCR. Day et al. (1997) studied the effects of changes in posture on the GVS response and concluded that the vestibular response is organized to stabilize the body rather than the head in space. Appendicular muscles are innervated through vestibulospinal tracts originating from the lateral vestibular nuclei. Direct and indirect connections via spinal interneurons to motor neurons of extremities have been found in animal studies (Lund and Pompeiano 1968; Shinoda et al. 1986). In humans, EMG response latencies of ~50–60 msec were found for appendicular vestibular reflexes (Britton et al. 1993; Fitzpatrick et al. 1994; Day et al. 1997; Ali et al. 2003; Son et al. 2008). These latencies are longer than expected for the presence of direct vestibulospinal connections and are consistent with the additional processes of postural gating and coordinate transformation associated with appendicular balance responses (Fitzpatrick and Day 2004). As discussed by Fitzpatrick and Day (2004), and as explored recently by Forbes et al. (2016), between immediate vestibular processing and regulation of balance there is a process of coordinate transformation from head‐in‐space to body‐in‐space and a process of gating or selection of biomechanically appropriate muscles. While previous work has established postural gating of the balance and not the cervical response, our contribution confirms the differential effect of perception of risk on balance, but not the cervical response.

In sum, axial and appendicular GVS reflexes were distinguished by several features. These include invariance of latency and magnitude of the response to fear of falling, and absence of cathodal acceleration at short latency. These different properties may reflect differences in innervation (medial vs. lateral vestibulospinal tracts) and different functional goals (cervical‐head stabilization vs. whole‐body balance).

### Does modulation of vestibular response with fear of falling depends upon the function of the reflex pathway?

Recently, authors have found that vestibular‐evoked myogenic potentials (VEMPs) in the neck (sternocleidomastoid) and soleus were increased marginally (9%, 12%) by height‐induced fear of falling, whereas other muscles including upper limb muscles were not enhanced by fear of falling (Naranjo et al. 2015). VEMPs are believed to arise predominantly from stimulation of the saccule (Rosengren et al. 2010). The saccule predominantly registers linear acceleration and pitch within the head‐defined sagittal plane (Fitzpatrick and Day 2004). Within the posture studied by Naranjo et al. (2015, 2016) stimulation of the saccule would evoke sensations of vertical and horizontal acceleration. An unanticipated horizontal acceleration would challenge balance (horizontal location of CoM relative to feet), and regulation of balance would require a response within muscles regulating horizontal location of CoM. A vertical acceleration would require a response to regulate vertical posture but would not challenge balance. The balance regulation system is sensitive to direction of threat (Mian and Day 2014; Forbes et al. 2016). Fear of falling would be expected to accentuate the response regulating balance and not the response regulating vertical posture. Naranjo et al. (2015) show precisely a general response unaffected by fear, and a response in muscles regulating horizontal translation of the head and body that is accentuated by fear, namely, soleus and sternocleidomastoid within their setup.

The GVS response arises from artificial vestibular feedback from the labyrinths (Fitzpatrick and Day 2004), which register rotation of the head in space. Head‐in‐space rotation requires a response to regulate the angle of head in space and, depending upon posture of the head relative to the feet, a response to regulate horizontal movement of the whole‐body CoM. Head rotation, per se (without translation), does not challenge balance, whereas horizontal movement of CoM does challenge balance. Hence, fear of falling would be expected to accentuate the balance response while the effect on the cervical‐head rotation response is more of an open question. Our results show a differential influence of fear of falling on the balance response to GVS as opposed to the cervical‐head rotation response.

Combined, our results and those of Naranjo et al. (2015, 2016) both support a thesis that vestibular feedback gain of balance responses is accentuated by fear of falling, and both support a thesis that modulation of response depends upon the function of the reflex pathway. Therefore, our results and those of Naranjo et al. (2015, 2016) contradict the thesis of a common central mechanism where fear of falling influences all vestibular feedback mechanisms (Naranjo et al. 2016).

### Implications for fear of falling

Clinically, important questions are the extent and mechanisms by which balance responses are influenced by fear (van Dieen et al. 2015). Our findings show that fear influences vestibular balancing reflexes. However, it is important to note that while fear of falling increases the gain of this balance reflex, it remains undetermined whether this leads to an increase or decrease in the risk of falling in the general population, and in elderly persons with a persistent fear of falling. Efficient balance control enables mobility. Hence, future studies could investigate whether the effect fear of falling on vestibular reflexes increases or decreases mobility in the general population, and in the elderly population in particular. Additionally, the asymmetric decline in sensory and vestibular function with aging may leave individuals vulnerable to the influence of fear on vestibular processing (Horak et al. 1989; Baloh et al. 1993; Kristinsdottir et al. 2000). Patient‐specific identification of the origin of balance performance decline is required and follow‐up studies with elderly persons and clinical subgroups could clarify mechanisms relating fear of falling to balance and mobility.

## Conclusion

To our knowledge, this study provides the most detailed full‐body kinematic analysis of the GVS‐evoked response to date. We parsed the whole‐body response (CoM) into its component parts (cervical, axial trunk, short‐, and medium‐latency lower extremity) and assessed the effect of fear of falling on each component. Results demonstrated the ability of kinematic analysis to reveal small responses, believed marginal through EMG, and also demonstrated opposing responses cancelling their effect within centre of mass and force plate data. These new data justify a hypothesis that short‐ and medium‐latency reflexes comprise a coordinated balance response. Results also indicated that fear differentially accentuates the appendicular balance response without influencing the axial vestibulocollic reflex.

## Conflict of Interest

The authors declare no conflicts of interest.

## Data Accessibility

## Supporting information




**Appendix S1.** Averaged GVS response.

**Simulation 1.** Effect of short‐latency moment on inverted pendulum CoM.

**Simulation 2.** Effect of short‐latency moment on upper body across flexible hip.

**Simulation 3.** Effect of short‐latency moments on upper body across flexible knee.

**Simulation 4.** Effect of short‐latency moment on upper body across flexible lower‐limb.

**Simulation 5.** Effect of neck moment on head and body below the neck.

## Appendix 1

### 1.1

The video Appendix S1 shows the mean GVS response of all participants, in the ground and height conditions. Comparable to Figure 7, mediolateral movement of the body nodes is shown. Dots and stick figures show mediolateral displacement of the head, trunk, and lower extremity body nodes with respect to the position at GVS onset. The left side represents the cathode side and the right side represents the anode side. Arrows represent mediolateral acceleration. Internode distances are not scaled. We recommend to use Quicktime 7 to play the video, as this program allows to skip through the video frame by frame using the left and right keys.

## Appendix 2 Link model simulation of response generated at short latency (SL)

### Aim

The aim of this modeling is to demonstrate, in the simplest way possible, the effect of a flexible lower extremity on the generation of anodal sway of the CoM at short latency.

### Outline

- Simulation 1, for reference, shows the effect of a transient moment, acting at short latency, across an ankle joint on a rigid single segment body. The transient moment produces an anodal sway of the whole body.

The simplest way to add flexibility to a lower limb is to add a single joint, for example, at a hip or at a knee location.

The simplest way to apply a moment to the upper body relative to the ground is to apply a moment of equal magnitude and direction to each joint (e.g., ankle and hip). This pair of moments cancels internally and acts externally on the upper body, relative to the ground.

- Simulation 2 shows the effect of a transient moment, at short latency, on the upper body across a lower extremity flexible at the hip.
- Simulation 3 shows the effect of the same transient moment, on the upper body across a lower extremity flexible at the knee.

Simulation 2, compared with Simulation 1, shows that flexibility at the hip results in cathodal buckling centered at the hip, and a small cathodal sway of the CoM preceding the main anodal sway.

Simulation 3, compared with Simulation 1, shows that flexibility at the knee results in cathodal buckling centered at the knee at short latency, preceding anodal sway of the CoM.

We note that the pattern of movement observed experimentally (Figs. 7 and 6) contains elements of both Simulations (2 and 3), but is closer to Simulation 3 on account of cathodal buckling centered at the knee. While mediolateral rotation at the level of the knee is not obvious, we should consider that these movements are at a millimeter scale and in the real human arise from two legs (Fig. 7). In support of Simulation 3, we also note that GVS in the head forward configuration is associated with a short‐latency response in the gastrocnemius muscle, which crosses the ankle and knee joints. If the goal of the response to GVS is to move the centre of mass, we further note that applying a moment on the upper body across a flexible knee has more effect on the centre of mass than applying a moment on the upper body across a flexible hip (c.f. video of Simulation 3 vs. Simulation 2).

We considered whether there was a simple way of combining flexibility at a knee and hip to reproduce more closely the experimental results shown in Figures 6 and 7.

The simplest way to apply a moment to the upper body relative to the ground across two flexible segments is to apply a moment of equal magnitude and direction to each joint (e.g., ankle, knee, and hip). This triplet of moments cancels internally and acts externally on the upper body, relative to the ground.

To alter the balance of flexibility at knee and hip joints to reproduce the experimental pattern, we increased the stiffness of the hip joint by a factor of 5 from the default value described below.

- Simulation 4 shows the effect of the same transient moment, on the upper body across a lower extremity flexible at the knee and hip, but with greater stiffness at the hip.

Simulation 4, compared with Simulation 1, shows that flexibility at the knee and hip, with this ratio of stiffness, results in cathodal buckling centered at the knee, and cathodal acceleration of the CoM preceding a main anodal sway of the centre of mass. The timing and magnitude of cathodal accelerations of the knee and hip match approximately the larger magnitude acceleration of the knee relative to pelvis and earlier timing of the peak cathodal pelvis acceleration relative to knee, all of which were observed in the experimental data (Fig. 6).

The reader may wonder whether the vestibulocollic reflex (VCR) can generate cathodal buckling in the lower extremity. To show the effect of a neck moment and to show that the VCR cannot generate cathodal buckling in the lower extremity, we added joints at vertebral levels C7 and L3 and thus divided the head and trunk segment into lumber, thorax, and head segments.

- Simulation 5 shows the effect of the same transient moment, generated at the neck joint (C7) acting on the head relative to the thorax.

Simulation 5 shows that a transient moment acting across the neck causes head lateral flexion relative to the thorax. This neck moment causes anodal acceleration of the head, and anodal acceleration of the body below the neck closing the angle at the neck. Anodal acceleration of the body below the neck is constrained by contact with the ground. The result is anodal buckling of the body below the neck. The exact profile of the anodal buckle depends upon the distribution of mass and joint stiffness in the body below the neck.

### Conclusion

These simulations demonstrate that a combined ankle–knee–hip moment at short latency provides anodal sway of the whole‐body centre of mass, very similar to, and appropriate to, the established goal of the medium‐latency response which is regulation of horizontal CoM location (balance). The cathodal buckling pattern of a lower leg is a mechanical byproduct of generating anodal sway of the whole‐body CoM.

### Simulation Methods

#### Mechanical system

This analysis uses a previously published model, which represents the standing human as a three‐segment model with a head and trunk segment (HAT), a thigh and a shank segment (Gawthrop et al. 2015; Loram et al. 2015, 2016). Mechanical segment properties (link mass, length, centre of mass location, radius of gyration) are taken from Winter (2009). To simplify exposition of effects we used passive springs of 100% gravitational stiffness, which by definition eliminates the gravitational effect on segment rotations from the vertical (Gawthrop et al. 2015; Loram et al. 2015, 2016). All simulations began with zero link angle from the vertical and zero velocity for all segments. We confirm that within the 0.33 sec simulation duration, variation in passive stiffness within physiological limits 20–100% (Kiemel et al. 2008) left the demonstration points unaltered. For simulation five mechanical properties were also taken from Winter (2009) and passive springs of 100% were used for all joints.

#### Control action

A transient moment was generated at short latency. To reproduce the timings of action at short latency a stimulus was applied as a 10 msec duration, burst of EMG, starting at 50 msec (Fig. 10A). EMG was translated to joint moment (Fig. 10A) using a second‐order linear transfer function, 1/(T_c_^2^ + 2T_c_ + 1), with time constant *T*_*c*_ of 70 msec and a delay of 15 msec giving an impulse of 0.25 Nms and peak moment of approximately 1 Nm (Bawa and Stein 1976). In each simulation, this impulsive moment was applied to each of the joints specified, with equal magnitude and clockwise direction looking into the figures.

To simplify exposition of effects, simulation results show the open loop effect of this action generated at short latency. To isolate the effect of the action generated at short latency, a medium‐latency EMG response starting at 100 msec was also excluded. We confirm that inclusion of time delayed (0.1 sec), continuous optimal feedback control (Gawthrop et al. 2015; Loram et al. 2015, 2016) was investigated, and excluded from this illustration as the demonstration points were unaltered.
